# Exposure and risks from wearing asbestos mitts

**DOI:** 10.1186/1743-8977-2-5

**Published:** 2005-10-03

**Authors:** John W Cherrie, Matthew Tindall, Hilary Cowie

**Affiliations:** 1 Institute of Occupational Medicine, Research Park North, Riccarton, Edinburgh, EH14 4AP, UK; 2 University of Aberdeen, Department of Environmental and Occupational Medicine, Foresterhill Road, Aberdeen, AB25 2ZP, UK; 3 Rilmac (Insulation) Ltd, Crofton Drive, Lincoln, LN3 4NJ, UK

## Abstract

**Background:**

Very high fibre inhalation exposure has been measured while people were wearing personal protective equipment manufactured from chrysotile asbestos. However, there is little data that relates specifically to wearing asbestos gloves or mitts, particularly when used in hot environments such as those found in glass manufacturing. The aim of this study was to assess the likely personal exposure to asbestos fibres when asbestos mitts were used.

**Results:**

Three types of work activity were simulated in a small test room with unused mitts and artificially aged mitts. Neither pair of mitts were treated to suppress the dust emission. The measured respirable fibre exposure levels ranged from <0.06 to 0.55 fibres/ml, with no significant difference in fibre exposure between aged and unused mitts. The use of high localised ventilation to simulate convective airflows from a furnace reduced exposure levels by about a factor of five. Differences between tasks were statistically significant, with simulated "rowing" of molten glass lowest and replacement of side seals on the furnace highest. Estimated lifetime cancer risk from 20 years exposure at the upper end of the exposure range measured during the study is less than 22 per 100,000.

**Conclusion:**

People who wore asbestos mitts were likely to have been exposed to relatively low levels of airborne chrysotile asbestos fibres, certainly much lower than the standards that were accepted in the 1960's and 70's. The cancer risks from this type of use are likely to be very low.

## Background

Asbestos protective clothing was widely used in "hot" industries such as foundries, steel plants and glassworks, and by fire fighters. Undoubtedly the use of such clothing has saved many lives and made the working conditions of others more bearable. Use of asbestos protective clothing was considered acceptable in the UK until the late 1970s and, for example, it was not until 1976 that Scottish health civil servants advised the fire service of the possible hazards posed by asbestos equipment used by fire fighters [1]. At that time it was concluded that although the risks to health were minimal, fire brigades should phase out their use and find replacement gloves made from alternative materials.

Bamber and Butterworth [2] first published data on the airborne fibre exposure while wearing asbestos protective clothing. They carried out a laboratory study where personal monitoring was undertaken on a subject wearing an asbestos apron and gauntlets while carrying objects and doing bench work. The laboratory was well ventilated with approximately 10 air changes per hour extracted at ceiling level. The airborne fibre concentrations measured in their six tests ranged from 2.4 to 4.2 fibres/ml, with a mean airborne fibre concentration of 3.5 fibres/ml.

In a later study by Gibbs [3], personal airborne fibre exposure from wearing asbestos safety coats, hoods, gloves and leggings was measured for workers at two ore reduction plants. The men working on furnaces prepared channels along which molten iron flowed, tapped the furnaces and kept the channels clear of slag during casting. They wore asbestos safety coats for the duration of the castings (0.5 to 1.25 hours), which were repeated at approximately 4-hour intervals. Asbestos gloves and hoods were also worn at times during the work. A second survey was undertaken at a small plant manufacturing elemental phosphorous where four men carried out work similar to that at the steel works. Asbestos safety coats and leggings were worn throughout the slagging operations but hoods and gloves were worn only when necessary. Personal monitoring at both plants was only carried out from the start to finish of the slagging operations (15 – 47 minutes).

At the steel works, the mean airborne fibre concentration measured during the 39 personal monitoring tests was 2.0 fibres/ml, with a range from 0.3 to 5.0 fibres/ml, based on a mean sampling period of 52 minutes. The analysis of these data suggested that the fibre release increased with age of the garments up to 8 weeks, although the number of measurements was too small and the correlation coefficient too low to reliably predict fibre release from garments of different ages. At the phosphorous manufacturing plant, the mean personal airborne fibre concentration measured by personal sampling was 14 fibres/ml. This was based on 6 tests and a mean exposure period of 35 minutes. The measured airborne fibre concentrations ranged from 9.9 to 26 fibres/ml. The reason for the considerable differences in measured airborne fibre concentrations between the two plants was not known, although Gibbs suggests the higher levels were because the coats and mittens in the phosphorous plant were untreated (i.e., not aluminized outside or dust suppressed) and leggings were also quite badly damaged.

One other possible contributor to the differences in measured airborne fibre concentrations between the two plants was the room volume. Although the exact volume of each workroom is not given, the steel plant is described as large in comparison with the small phosphorous manufacturing plant. Damaged asbestos clothing could have contaminated the workplace and workers may then have disturbed this contamination. The lower airborne fibre concentrations were measured in the larger plant, which may be explained by the dilution effect of general ventilation being greater in large rooms in comparison with smaller ones [4].

Riediger [5] describes a combined controlled laboratory test and associated factory study of fibre release from asbestos clothing. He showed that in the laboratory tests asbestos cloth impregnated with a binder could produce airborne fibre concentrations that were approximately four times lower than those generated by untreated cloth. However, only three of the five treated asbestos cloths were effective and the other two samples produced higher fibre levels than any of the untreated materials. Heating impregnated asbestos cloth at 200°C reduced the effectiveness of the binder.

A study of occupational exposure to airborne fibres from the use of asbestos gloves was published by Samimi and Williams [6]. They investigated fibre exposure during the simulation of laboratory procedures in an unventilated isolation chamber and in a biology preparation room, as well as during actual work carried out by laboratory staff in two separate situations. The laboratory tasks where asbestos gloves were used comprised routine sterilization and the drying of laboratory glassware, both of which required the workers to put their hands in a hot autoclave or oven. The asbestos gloves were classified into four categories based on structural integrity and apparent surface cleanliness: well-worn & clean, well-worn & lightly soiled, well-worn & heavily soiled and brand-new. All gloves were of the same type. The experimenter carried out the simulation of work activities inside the isolation chamber after inserting his arms through two portholes in the front panel. The same sterilization operation was also simulated in a well-ventilated biology preparation room that had five air changes per hour. The interval between consecutive operations was either 30 minutes to represent the normal workload, or 10 minutes to represent a heavy workload. In the studies performed on workers in their actual workplaces, air samples were collected from the breathing zone of each worker and 75 cm above the tabletop where the gloves were laid or tossed.

The mean time weighted average (TWA) concentrations of airborne fibres from the 176 measurements in the isolation chamber, ranged from 0.95 to 12 fibres/ml. The minimum TWA concentration measured was 0.61 fibres/ml for well-worn and heavily soiled gloves. The maximum TWA concentration measured was 16 fibres/ml for well-worn and clean gloves. The results showed that clean well-worn gloves emitted significantly more fibres than did brand-new gloves, but fibre emission decreased with increased surface soiling.

Eighty air samples were collected during a simulation carried out in the well-ventilated laboratory. The range of mean TWA airborne fibre concentrations was 0.07 to 0.99 fibres/ml for the personal samples, and 0.06 to 0.60 fibres/ml for the static samples. These airborne concentrations were considerably lower than those obtained in the isolation chamber. This was due to the dispersion of fibres within the larger volume of the room, as well as the fact that the room was well ventilated when compared to the unventilated isolation chamber.

Thirteen samples were collected by Samimi and Williams in actual workplaces. The maximum and minimum TWA airborne fibre concentrations ranged from 0.07 to 2.93 fibres/ml for personal samples and from 0.04 to 0.74 fibres/ml for static samples (sampling over an 8-hour shift). With this limited number of samples, it was found that exposure levels depended more on the particular laboratory than on glove condition or workload, which were the main influencing factors under the controlled conditions of the simulation experiments. For example, when comparing the fibre exposures from the same task carried out with the same glove type, but at different laboratories, one was found to be 29 times higher than the other. This was explained by the presence of an efficient exhaust system over the row of five autoclaves in the laboratory where the lower exposures were measured.

As is clearly demonstrated by these studies, there is considerable room for debate over the level of fibre exposure from wearing asbestos protective clothing. Gibbs [3] in his study recorded a maximum airborne fibre concentration of 26 fibres/ml from personal sampling during slagging operations in a phosphorous manufacturing plant, whilst Bamber and Butterworth [2] and Samimi and Williams [6] in their studies measured airborne fibre levels which were generally between 1 and 5 fibres/ml.

Tougher legislation and greater awareness of the risks of working with asbestos have ensured that most organisations in Europe no longer use products containing asbestos, and many are in the process of eliminating all sources of asbestos from their work environments. However, as well as organisations becoming more aware of the risks of asbestos exposure, workers' knowledge of such risks has also increased. This increased awareness has resulted in more civil claims for compensation being made against employers for previous asbestos use. In many situations where asbestos exposure took place there is limited data on which to assess the likely airborne fibre concentrations from past working conditions. One approach to obtain more reliable information is to simulate work activities undertaken in the past and measure fibre exposure.

The aim of this study was to assess the personal exposure to airborne fibres arising from the use of chrysotile asbestos mitts worn in a glass manufacturing plant. This information was then used to assess the likely health risks to workers who had worn asbestos mitts.

## Results

**Figure 1:**
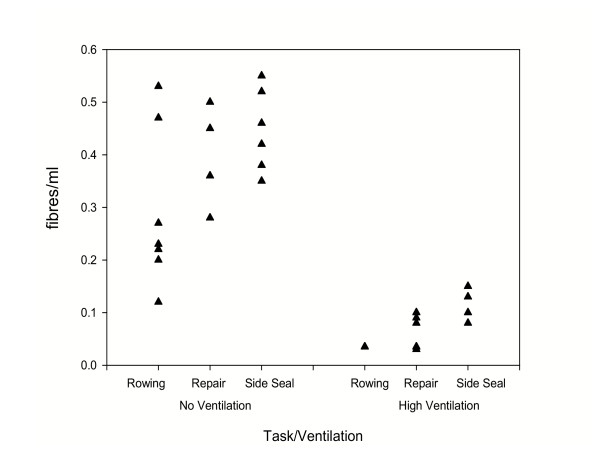
Fibre exposure levels during simulated work with asbestos mitts.

The airborne fibre sampling results from 33 personal samples collected during the simulation of the three different tasks under the various work conditions are summarised in Figure 1. In the figure each point represents the average fibre concentration measured during the activity. The mean personal airborne fibre concentrations for each test condition ranged from 0.03 to 0.35 fibre/ml for rowing, 0.05 to 0.48 fibres/ml for glass window repair and 0.09 to 0.47 fibres/ml for side seal replacement. The lowest average personal fibre concentrations were all obtained when high localised ventilation was used, whereas the maximum mean concentrations were measured for all three tasks when no ventilation was used. This suggests that the presence of localised ventilation substantially reduced the airborne fibre concentrations. This trend was also shown by both the 1 metre and 3 metre static area samples (data not presented in this paper). The measured airborne fibre concentrations for both the glass window repair and side seal replacement tasks were also generally higher than those for the rowing.

An analysis of variance was carried out on the data to investigate the differences between the three factors: type of gloves (unused versus aged), task and whether ventilation was used. The results showed the decrease in exposure levels when ventilation was used was highly statistically significant. Differences observed between the mean airborne fibre concentrations for the three simulation tasks were also highly significant (p < 0.01). Differences between mean airborne fibre concentrations for aged gloves and unused gloves were not significant. There were no significant interaction effects for the three factors (e.g. relative differences between tasks were similar whether or not ventilation was used).

The mean heart rate for each of the three simulation tasks ranged from 94 to 133 beats per minute. These data showed that the most strenuous task was the side seal replacement, which would be classed as "very heavy work" (estimated breathing rate 37.5 – 50 l/min). Both rowing and glass window repair were classed as "moderate" activities in terms of severity of workload (breathing rate 12.5 – 25 l/min). Glass window repair was the least strenuous activity with the lowest mean, maximum and minimum heart rate values.

## Discussion

There was no difference between the measured airborne fibre concentration when unused or aged asbestos mitts were used to carry out the various tasks. This is contrary to what was observed in other studies and the most likely explanation is a combination of ineffective artificial ageing of the mitts and the abrasive nature of the tasks carried out in this study. Observations made during the tests showed that it was the abrasion of the mitts on sharp metal edges that resulted in obvious release of airborne dust and this would have applied equally to both types of glove.

We have shown that the use of a high level of localised ventilation significantly reduced the measured airborne fibre concentration when compared with the results from the same simulations carried out without any ventilation. The ventilation was designed to simulate the upwards flow of air produced by thermal convection from a hot glass float bath and the results show that the presence of hot work equipment would probably have reduced the workers exposure. From the present data the exposures for those working next to a glass float bath were likely to have been about a fifth of what they would have otherwise have been.

Examination of the results from the three different tasks shows that the side seal replacement and glass window repair tasks generally created similar airborne fibre concentrations (0.05 to 0.48 fibres/ml), whilst the rowing task produced lower airborne fibre concentrations (0.03 to 0.35 fibres/ml). These differences may be explained by the nature of the work. Both the side seal replacement and glass window repair required the subject to grip the object along narrow edges, some of which were sharp and this was seen to generate visible dust emission from the glove. The rowing task did not involve handling any sharp abrasive edges. The 2 m long steel pole used for rowing was cylindrical and had a smooth surface. Nevertheless, there was slight abrasion on the surface of the mitts when carrying out this task, as the hands had to rotate the steel pole in the hand whilst moving the pole backwards and forwards through the window.

There have been a few studies published on asbestos fibre exposure where asbestos protective clothing or mitts were the only source of exposure [2,3,5,6]. In previous research it was not been possible to determine the relative contribution of each item of asbestos protective clothing to the overall airborne fibre exposure. This makes it difficult to directly compare the results from these studies with the results from our research involving only asbestos mitts. However, it seems likely that much of the difference between these earlier studies and our simulations arises from the poor condition of the clothing worn in the workplace studies.

The only directly comparable study to ours is that published by Samimi and Williams [6]. This study investigated airborne fibre exposure in a biology preparation room and during work at two other laboratories. The range of mean airborne fibre concentrations was 0.07 to 0.99 fibres/ml for the personal samples. Thirteen samples were collected at the other workplaces where the minimum and maximum personal airborne fibre concentrations were 0.07 and 2.93 fibres/ml.

It is unclear why personal fibre exposures measured in the Samimi and Williams study were higher than those measured by us, as the work activities carried out in this earlier study were probably less damaging on the integrity of the gloves, when compared to the tasks carried out in our study. One reason may have been that the gloves used by Samimi and Williams show were generally in a poorer state in terms of structural integrity than the mitts that we used. Another reason that affects all of the historic studies is the poorer standard of quality assurance employed in studies carried out in the past compared with that used routinely today. This was highlighted in a paper published by Gibbs *et al *[7], which showed large intra-laboratory differences in fibre counting results. This lower standard of quality assurance in earlier work could possibly have resulted in either the over-estimation or under-estimation of fibre exposure in studies published at that time.

The results from our simulation study clearly show that tasks undertaken by glass furnace workers whilst wearing asbestos mitts would have resulted in asbestos fibres being released into the air. However, the contribution of wearing asbestos mitts to overall personal exposure to airborne asbestos fibre was probably quite low. There are a number of reasons for this assumption. Firstly, the simulation conditions that most accurately represented the actual workplace were those where high localised ventilation was used. This ventilation mimicked the upwards flow of air created by thermal convection next to a bath of molten glass, and if anything, was probably less than would normally be encountered. The results of personal monitoring for all three tasks under these conditions were very low, with the measured airborne fibre concentrations below the analytical detection limit of the method, for both the glass window repair and rowing, with the results for side seal replacement ranging from 0.09 to 0.14 fibres/ml.

Secondly, for the purposes of our study, the subject carried out each simulation over a 30-minute period, continuously repeating during that period one of the three tasks being investigated. This was necessary to generate an airborne fibre concentration that could be accurately evaluated by the analytical method. However, this regime of work was not employed in the past in the workplace. The furnace man would typically carry out one of the three tasks on an intermittent and random basis, whereas in the simulation the tasks were repeated about 100 times during the experiment. Although justified for the purposes of the study, the method has probably generated airborne fibre concentrations that were higher than those likely to be produced by wearing asbestos mitts in the workplace.

Thirdly, the relatively small volume of the experimental enclosure may have increased exposures over what would have occurred in the past. The dilution effect of general ventilation is usually greater in large areas in comparison with smaller areas and this could result in a lower measured airborne fibre concentration in the larger area next to the glass float bath, when all other influencing factors are the same in both the large and small areas. The experiments do not take account of exposure from residual asbestos contamination in a workplace resulting from fibre release from damaged clothing, but this type of secondary source would generally be small in comparison with direct emission. Other sources might however predominate in some circumstances, e.g. where other asbestos-containing materials were disturbed.

It seems unlikely that glass float furnace men wearing chrysotile asbestos mitts would have been exposed to respirable concentrations of asbestos above the present UK control limit for chrysotile. During the 1960's and 70's the standards that were accepted by the scientific community were higher than currently applied, although they mostly reflected concerns about non-malignant disease. In 1968 the British Occupational Hygiene Society published an internationally recognised hygiene standard for chrysotile asbestos dust [8,9]. This standard implied that the risk of having early clinical signs of asbestos-related disease would be less than one percent for 50 years exposure at 2 fibres/ml. Even working continuously carrying out the dustiest activity, workers wearing asbestos mitts in a glass manufacturing plant could never have received such an exposure.

Other workers who wore asbestos gloves or mitts, e.g. firemen or laboratory workers, would probably have had higher exposure than the glass workers because they would not necessarily have been working close to high convective airflows. In these situations the measurements we have made without ventilation might provide the best estimate of exposure level from wearing asbestos mitts, i.e. about 0.5 fibres/ml while the gloves were worn (see Figure 1).

Hodgson and Darnton from the UK Health and Safety Executive have carried out an extensive review of epidemiological studies that inform the quantitative link between cancer risks and asbestos exposure [10]. They provide mathematical models linking cumulative exposure to asbestos with both lung cancer and mesothelioma; in both cases the models are non-linear functions dependent on the cumulative inhalation exposure to asbestos, although for mesothelioma the risk is calculated separately for pleural and peritoneal tumours. The risk of mesothelioma increases as the time since first exposure increases and Hodgson and Darnton allow for this by using age-related adjustment factors.

We have used these models to estimate the risks for a glass worker aged 20 when first employed (in 1955) who worked for 20 years using chrysotile mitts. Assuming he was exposed for 10 minutes at the estimated 90^th ^percentile for each task each day his annual average exposure would have been 0.012 fibres/ml. In this calculation we have weighted the side seal replacement task three times more than the others to account for the higher breathing rate during this work. Based on these assumptions the best estimate of his lifetime risk of mesothelioma is around 3 in 100,000 and the risk of lung cancer is less than one per 100,000. The "highest arguable" risks (as defined by Hodgson and Darnton) were 16 per 100,000 for mesothelioma and 6 per 100,000 for lung cancer, which would equate to a total annual risk of about 3.7 per million. Even these highest estimates are around the risks that most would consider trivial, i.e. around 1 in a million per year.

The estimates are prone to uncertainty because of the processes involved in estimating the actual exposure of someone wearing asbestos mitts, from the analysis used to quantify the association between exposure and cancer risk and from the necessity to extrapolate this relationship to low exposures, certainly lower than most asbestos workers would have experienced in the past. However, despite this we believe that our measurements show that wearing asbestos mitts would have given rise to relatively low cumulative exposures to chrysotile asbestos, and taking account of the possible uncertainties in the process the risk of death from cancer from such exposures must be low; we believe trivially low.

## Conclusion

In the past protective mittens made from chrysotile asbestos were commonly used in glass manufacturing and fibres were released from asbestos mitts while they were being worn. During simulated work activities the airborne concentration in the workers breathing zone did not exceed 0.5 fibres/ml. Lower concentrations were measured in environmental conditions designed to reproduce high localised convective airflows found in glass plants. The lifetime risk of a worker contracting mesothioma or lung cancer from 20 years of past use of asbestos mitts in the glass industry was estimated to be 22 per 100,000, which is very low.

## Methods

The tests were carried out in an asbestos enclosure designed and constructed to the standard recommended in the Health and Safety Executive [11]. The size of this enclosure was approximately 45 m^3^, with dimensions 5 m × 3 m × 3 m. Extract ventilation was provided by a fan and a high-efficiency particle arrester (HEPA) filter. Air was extracted from the enclosure at ceiling level through a canopy, located directly above the workstation, and carried via flexible ducting to the extraction unit where the air was filtered. Again using flexible ducting, the filtered air was carried back into the enclosure and discharged upwards from an elevated 1 m × 1 m platform, upon which the test subject stood.

The workstation in the enclosure was to be used to simulate activities carried out close to a hot glass tank where upward convective airflows are found. The extraction system was designed to provide an upward air velocity of approximately 3 to 4 m/s. It was not possible during the course of this study to measure the upward convective airflow next to a hot glass tank in a glass works, although it was assumed that such airflow could be quite high.

This study investigated the three most common tasks where asbestos mittens were reportedly used in glass production plants. These tasks were:

1. *"Rowing" of molten glass. *This task was simulated using a window taken from a float bath and an approximately 2 m long steel pole that was used to move glass in a bath. Half of the steel pole was placed through the window situated at chest height, with the other half being gripped firmly and moved in a 'rowing' motion.

2. *Removal and replacement of a glass window in a float bath. *This task required the loosening of clamps holding the window in its frame, removing the window, setting it aside and then replacing it.

3. *Removal and replacement of a side seal in a float bath. *This task was simulated by lifting the side seal from the floor next to the workstation, pushing it into an orifice at chest height, and then removing it and placing it back on the floor.

Each of the three tasks was simulated for a period of 30 minutes. Throughout the test the subject's heart rate was monitored continuously using a "Polar Sport Tester" heart rate monitor, which recorded the subjects heart rate at 60 second intervals. This data enabled the subjects breathing rate to be estimated [12]. These data were used to make an assessment of breathing rate during the tasks.

A glass company provided two pairs of chrysotile asbestos gloves, made in the 1970's, for the exercise. One pair were unused and still in the original packaging, whilst the second pair had either been unused or had had very light usage. The tests were undertaken separately with the unused mitts and with the second pair artificially aged by heating the gloves for 20 hours at 100°C followed by hammering them in sealed packaging for five minutes inside an enclosed glove box.

The measurement of both personal fibre exposure and airborne fibre concentrations within the test room were made [13]. Two personal samples were collected for each simulation exercise. The sampling heads were positioned in the test subject's breathing zone, i.e. within approximately 200 mm of the nose and mouth, one on each side of the head. Prior to sampling the flow rate was set at 2.0 l/min, and this was checked both during and after the sampling period using a calibrated flow meter. In addition to the personal sampling, two static room samples were collected during each simulation exercise, one in the test subject's near-field (i.e. within 1 m of the breathing zone) and another in the far-field (i.e. approximately 3 m distant from the workstation). Both static samples were collected as described for the personal samples, except the sampling flow rate was set at 8.0 l/min. Both the 1 m and 3 m sampling heads were situated approximately 1.5 m from the ground. The results from the static samples are not presented in this paper.

Twelve separate tests were undertaken during the simulation exercise involving various combinations of the three tasks, the two types of mitt (used and unused) and two ventilation conditions (none and high). In addition, five of the tests were carried out twice to assess the repeatability of the measurements. During all of the tests the subject wore a high efficiency positive pressure respirator and protective clothing.

After each simulation the test enclosure was thoroughly cleaned using a high efficiency vacuum cleaner. Wet wipes were used to remove all traces of asbestos dust or debris produced during the simulation exercise. Air monitoring was undertaken after the cleaning of the test enclosure to ensure that the airborne fibre concentration was below 0.010 fibres/ml.

All the membrane filters were analysed using procedures complying with the United Kingdom Accreditation Service (UKAS) by an experienced analyst using the HSE method MDHS 39/4 [13].

Statistical analysis of the data was undertaken using a general linear model to assess the significance of the three different factors which were expected to influence the measured airborne fibre concentrations: glove type (i.e. unused or aged), ventilation condition (i.e. none or high localised ventilation) and simulation task (i.e. Rowing, Glass window repair or Side seal replacement). This approach was used because only five of the twelve tests were repeated. This produced an unbalanced data set that did not allow a simple analysis. The three factors were analysed using an Analysis of Variance test, to test all the factors both independently from each other, as well as for any interactions that were taking place between them.

Cancer risks from asbestos exposure were estimated using the method described by Hodgson and Darnton [10]. The percent excess mortality from mesothelioma (*P*_*m*_) was estimated using equation 1.

*P*_*m *_= *A*_*pl*_*X*^*r *^+ *A*_*pr*_*X*^*t *^    *(equation 1)*

where *A*_*pl *_and *A*_*pr *_are constants of proportionality for pleural and peritoneal risks, *X *is the cumulative exposure (in fibres/ml.years) and *r *and *t *are the slopes of the exposure-response on log-log scales.

Two sets of coefficients were used: one for the "best estimate" of risk and the other for the "highest arguable" risk estimates. An adjustment factor was used to allow for the age at which the person was first exposed, as described by Hodgson and Darnton.

Lung cancer excess percent excess mortality was similarly estimated using equation 2.

*P*_*L *_= *A*_*L*_*X*^*r *^    *(equation 2)*

where *A*_*L *_is the lung cancer constant and *r *is the slope of the exposure response on log-log scales (note the coefficient *r *in this equation is different from that in equation 1).

These estimates are based on British male mortality in 1997 when 9.5% of deaths were from lung cancer. The predictions therefore represent the past smoking prevalence of older men. Non-smokers would have substantially lower predicted risks and lifetime smokers would have lung cancer risks about double those quoted.

Calculations were undertaken using an EXCEL spreadsheet supplied by the authors (Hodgson, personal communication).

## Competing interests

JWC and HC prepare reports in connection with various asbestos civil litigation cases, some of which involve asbestos gloves or mitts.

## Authors' contributions

MT undertook the experimental work and assisted in the preparation of the manuscript. HC carried out the statistical analysis and the risk assessments. JWC conceived the project, supervised the work and prepared the manuscript. All authors read and approved the final text of the manuscript.
